# T‐Cell Immunoglobulin and Mucin Domain 1 (Tim1) as a Prognostic Factor Associated With Therapeutic Resistance in Human Breast Carcinoma

**DOI:** 10.1155/ijbc/3747828

**Published:** 2026-04-15

**Authors:** Mio Yamaguchi-Tanaka, Kiyoshi Takagi, Mai Sawafuji, Ai Sato, Kanoko Nakamura, Yasuhiro Miki, Minoru Miyashita, Takashi Suzuki

**Affiliations:** ^1^ Department of Pathology and Histotechnology, Tohoku University Graduate School of Medicine, Sendai, Miyagi, Japan, tohoku.ac.jp; ^2^ Department of Anatomic Pathology, Tohoku University Graduate School of Medicine, Sendai, Miyagi, Japan, tohoku.ac.jp; ^3^ Department of Breast and Endocrine Surgical Oncology, Tohoku University Graduate School of Medicine, Sendai, Miyagi, Japan, tohoku.ac.jp; ^4^ Department of Pathology, Tohoku University Hospital, Sendai, Miyagi, Japan, tohoku.ac.jp

## Abstract

**Background:**

Therapeutic resistance, including resistance to endocrine therapy in ER‐positive tumors and to chemotherapy in aggressive subtypes, remains a major clinical challenge in breast cancer. T‐cell immunoglobulin and mucin domain 1 (Tim1), a Type I transmembrane glycoprotein, has been reported to be aberrantly expressed in various cancer cells and contribute to tumor progression. However, its clinical significance in breast cancer and association with therapy resistance remain largely unclear.

**Methods:**

We investigated Tim1 expression by immunohistochemistry in 116 breast carcinoma tissues and analyzed its correlation with clinicopathological parameters and clinical outcomes according to chemotherapy and endocrine therapy status.

**Results:**

Tim1 immunoreactivity was detected in the cytoplasm and cell membranes of breast carcinoma cells but was negligible in the normal breast epithelium. Tim1 expression was significantly associated with pathological T factor, lymph node metastasis, histological grade, and Ki67 labeling index. Tim1 immunoreactivity was significantly correlated with an increased risk of recurrence, and multivariate analyses demonstrated Tim1 as an independent adverse prognostic factor for disease‐free survival. In addition, Tim1 remained correlated with the risk of recurrence in patients who had received chemotherapy or endocrine therapy.

**Conclusions:**

Tim1 might be an important therapeutic target for improving therapy in breast cancer patients and could be a strong adverse prognostic factor associated with therapeutic resistance.

## 1. Introduction

Breast cancer is the most commonly diagnosed malignancy in women worldwide and remains a leading cause of cancer‐related death [1]. Estrogen receptor (ER) is frequently expressed in breast cancer cells, and endocrine therapies targeting estrogen signaling have markedly improved outcomes in ER‐positive patients. Nevertheless, both intrinsic and acquired resistance to endocrine therapy remain major clinical challenges [2]. For more aggressive subtypes, such as triple‐negative breast cancer (TNBC), which lack therapeutic targets including ER and human epidermal growth factor receptor 2 (HER2), cytotoxic chemotherapy is widely used. However, resistance to chemotherapy is also a growing concern, with approximately 25% of patients developing distant metastases after adjuvant treatment [3]. These resistance mechanisms drive recurrence and poor prognosis, highlighting the urgent need for novel biomarkers and therapeutic targets to better predict or overcome therapy resistance.

T‐cell immunoglobulin and mucin domain 1 (Tim1), also known as hepatitis A virus cellular receptor 1 (HAVCR1), is a Type I transmembrane glycoprotein initially identified as a costimulatory molecule expressed on activated T cells [4]. Tim1 is a member of the Tim family, which also includes Tim3 and Tim4. Tim4, expressed on antigen‐presenting cells, functions as a ligand for Tim1, and their interaction plays a role in regulating T‐cell proliferation [5]. In T cells, Tim1 is crucial for modulating immune responses by promoting activation, proliferation, and cytokine production, particularly in Th2‐mediated immunity [6]. In addition, Tim1 recognizes phosphatidylserine (PS) on apoptotic cells, extracellular vesicles, and viral envelopes, thereby facilitating viral entry [7], and Tim1 expressed in epithelial cells contributes to the clearance of apoptotic cells [8, 9].

Recently, Tim1 has been reported to be aberrantly expressed in various types of cancer cells [10–17]. In many of these malignant tumors, Tim1 has been reported to contribute to tumor progression and poor patient prognosis by promoting cell proliferation, invasion, and epithelial–mesenchymal transition (EMT), as well as by regulating cytokine secretion. In breast cancer, although a previous study demonstrated that Tim1 is expressed in cancer cells and that its expression in breast cancer cells was not correlated with lymph node metastasis or stage by immunohistochemistry [18], the relationship between Tim1 and breast cancer treatment remains unclear. In addition, Tim1 mRNA in plasma extracellular vesicles has been suggested as a potential diagnostic marker for breast cancer [19]. Therefore, in this study, we investigated the clinical significance of Tim1 expression in breast cancer cells and its association with therapeutic resistance using immunohistochemistry on 116 breast carcinoma tissue samples.

## 2. Materials and Methods

### 2.1. Patients and Tissues

We analyzed 116 invasive ductal breast cancer specimens obtained from patients who underwent surgical resection at Tohoku University Hospital between 2007 and 2008. All tissues were fixed in 10% neutral buffered formalin and paraffin‐embedded. Clinical outcomes were evaluated using disease‐free survival (defined as the interval from surgery to locoregional or distant recurrence) and breast cancer–specific survival (defined as the interval from surgery to death caused by breast cancer). The median follow‐up times were 59 months (range, 3–84) and 61 months (range, 3–84), respectively. Among the patients, 59 received neoadjuvant or adjuvant chemotherapy, or both. Regarding endocrine therapy, 94 had ER‐positive breast cancer and received it, 2 had ER‐positive breast cancer without it, 2 had ER‐negative breast cancer with it, and 18 had ER‐negative breast cancer without it. This study was approved by the Ethics Committee of the Tohoku University School of Medicine.

### 2.2. Immunohistochemistry

Anti‐Tim1 antibody (clone 219211; R&D Systems, Minnesota, United States) was used at a dilution of 1:400. Antigen retrieval was performed in Tris/EDTA buffer (pH 9) using an autoclave at 121°C for 10 min. The antigen–antibody complex was visualized with the Histofine Kit (Nichirei Biosciences, Japan) and 3,3 ^′^‐diaminobenzidine, followed by hematoxylin counterstaining as previously described [20]. Human kidney tissue was used as a positive control.

### 2.3. Assessment of Immunoreactivity

The staining patterns were classified based on staining intensity and area, and cases with immunoreactivity in more than 10% of carcinoma cells were considered Tim1‐positive, based on the criteria previously used to evaluate immunostaining [21–23]. The immunohistochemical evaluation of ER, progesterone receptor (PR), HER2, and the Ki67 labeling index (LI) was performed according to previously published criteria [24].

### 2.4. Statistical Analysis

Statistical analyses of immunohistochemistry data were conducted using JMP Student Edition (SAS Institute, Cary, North Carolina, United States). Associations between clinicopathological factors and Tim1 immunoreactivity were assessed using the *χ* ^2^ test or the Mann–Whitney *U* test. Survival curves were generated using the Kaplan–Meier method and compared by the log‐rank test. Univariate and multivariate analyses were performed with the Cox proportional hazards model. A *p* value of less than 0.05 was considered statistically significant.

## 3. Results

### 3.1. Immunohistochemistry for Tim1 in Human Breast Carcinoma Tissues

**Figure 1:**
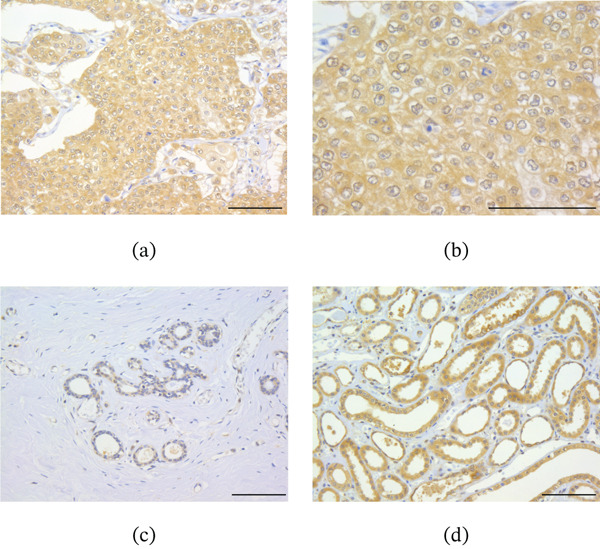
Immunoreactivity of Tim1 in human breast carcinoma tissues. (a–c) Tim1 immunoreactivity was observed in both the cytoplasm and membrane of breast carcinoma cells (a, 200×; b, 400×), with no staining detected in the normal breast epithelium (c). (d) The kidney was used as a positive control, showing Tim1 immunoreactivity in renal tubular epithelial cells. Bar = 100 *μ*m, respectively.

Tim1 immunoreactivity was detected in both the cytoplasm and cell membranes of breast carcinoma cells (Figure 1a,b), whereas it was negligible in normal breast epithelium (Figure 1c). In the positive control (kidney), Tim1 expression was observed in the uriniferous tubules (Figure 1d). Overall, 62 patients (53%) were classified as Tim1‐positive. The associations between Tim1 immunoreactivity and clinicopathological parameters are summarized in Table 1. Tim1 immunoreactivity was significantly correlated with pathological T factor (*p* = 0.008), lymph node metastasis (*p* = 0.013), histological grade (*p* = 0.0006), and Ki67 LI (*p* < 0.0001).

**Table 1:** Clinicopathological characteristics of Tim1 in breast carcinoma tissues (*n* = 116).

	Tim1		*p*
	Negative (*n* = 54)	Positive (*n* = 62)	
Age∗	55 (29–82)	57 (27–87)	0.36
Menopausal status			
Pre‐	23	18	0.13
Post‐	31	44	
pT			
pT1	43	35	**0.008**
pT2–4	11	27	
Lymph node metastasis			
Negative	43	36	**0.013**
Positive	11	26	
Stage			
1	37	30	0.086
2	11	19	
3	6	13	
Histological grade			
1	30	15	**0.0006**
2	19	27	
3	5	20	
ER			
Negative	7	15	0.12
Positive	47	47	
PR			
Negative	13	24	0.092
Positive	41	38	
HER2			
Negative	47	52	0.63
Positive	7	10	
Ki67 LI∗	7 (1–38)	16.5 (1–60)	**< 0.0001**

*Note:* Asterisk “∗” denotes that data were presented as median (minimum–max). All other values represent the number of cases. *p* < 0.05 was considered significant and described as bold.

### 3.2. Association Between Immunoreactivity of Tim1 and Clinical Outcomes in Breast Cancer Patients

**Figure 2:**
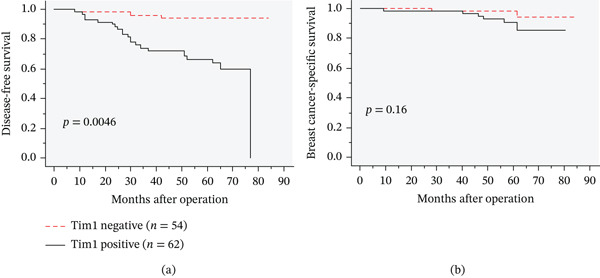
Prognostic analysis according to Tim1 immunoreactivity. (a) Disease‐free survival curves and (b) breast cancer‐specific survival curves according to Tim1 immunoreactivity in breast carcinoma cells (*n* = 116). Survival curves were generated by the Kaplan–Meier method and statistical analysis was performed using the log‐rank test.

We subsequently examined the relationship between Tim1 immunoreactivity and clinical outcomes, as assessed by disease‐free survival and breast cancer–specific survival. Tim1 immunoreactivity was significantly associated with shorter disease‐free survival (Figure 2a, *p* = 0.0046), whereas no significant correlation was observed with breast cancer–specific survival (Figure 2b, *p* = 0.16). The results of the univariate and multivariate analyses are summarized in Table 2. Univariate analysis identified Tim1, pT, lymph node metastasis, histological grade, ER, PR, and Ki67 LI as significant prognostic factors for disease‐free survival, whereas multivariate analysis revealed that only Tim1 (*p* = 0.0016) was an independent prognostic factor for disease‐free survival.

**Table 2:** Univariate and multivariate analysis of disease‐free survival in 116 breast cancer patients.

	Univariate		Multivariate	
	*p*	Relative risk (95% CI)	*p*	Relative risk (95% CI)
pT (pT2–4/pT1)	**0.0002**	4.5 (2.0–10)	0.080	2.9 (0.88–9.4)
Lymph node metastasis (positive/negative)	**0.0052**	3.1 (1.4–6.8)	0.65	1.3 (0.42–4.0)
Histological grade (3/1 + 2)	**0.012**	2.9 (1.3–6.6)	0.21	0.52 (0.19–1.4)
ER (negative/positive)	**0.012**	3.0 (1.3–6.8)	0.90	1.1 (0.35–3.3)
PR (negative/positive)	**0.0008**	4.0 (1.7–9.1)	0.15	2.3 (0.77–7.0)
HER2 (negative/positive)	0.16	2.5 (0.59–11)		
Ki67 LI (≥ 20%/< 20%)	**0.0001**	4.8 (2.1–11)	0.15	2.3 (0.74–7.2)
Tim1 (positive/negative)	**< 0.0001**	7.8 (2.3–26)	**0.0016**	5.5 (1.6–19)

*Note:* *p* < 0.05 was considered significant and examined in multivariate analysis and described as bold.

### 3.3. Association Between Tim1 Immunoreactivity and Clinical Outcomes in Breast Cancer Patients Treated With Chemotherapy and Endocrine Therapy

**Figure 3:**
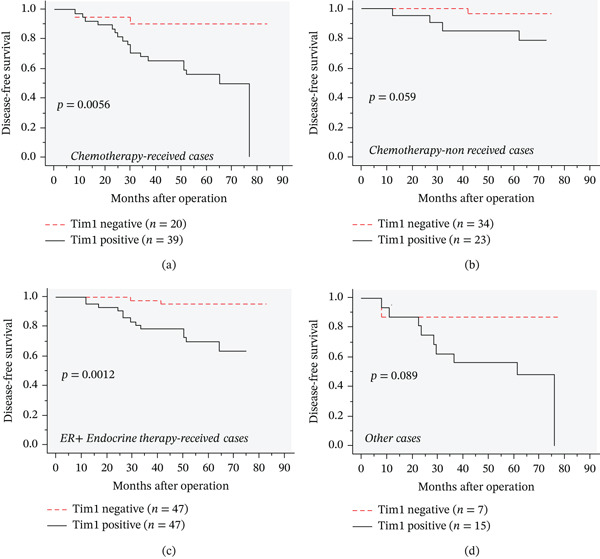
Prognostic analysis according to Tim1 immunoreactivity according to status of chemotherapy and endocrine therapy. (a, b) Disease‐free survival curves according to Tim1 immunoreactivity in breast carcinoma cells in breast cancer patients who had received (a, *n* = 59) or not received chemotherapy (b, *n* = 57). (c. d) Disease‐free survival curves according to Tim1 immunoreactivity in breast carcinoma cells of ER+ breast cancer patients who had received endocrine therapy (c, *n* = 94) or in all other cases (d, *n* = 22). Survival curves were generated by Kaplan–Meier method and statistical analysis was performed using log‐rank test.

When we analyzed the association between Tim1 and clinical outcomes according to chemotherapy status, Tim1 immunoreactivity was significantly correlated with shorter disease‐free survival in patients who had received chemotherapy (Figure 3a, *p* = 0.0056), whereas no significant correlation was observed in those who had not received chemotherapy (Figure 3b, *p* = 0.059). Similarly, in the chemotherapy‐treated group, Tim1 immunoreactivity, along with pT, PR, and HER2, was confirmed as an independent prognostic factor for disease‐free survival (Table 3; pT, *p* = 0.025; PR, *p* = 0.0051; HER2, *p* = 0.013; Tim1, *p* = 0.024).

**Table 3:** Univariate and multivariate analysis of disease‐free survival in 59 breast cancer patients who had received chemotherapy.

	Univariate		Multivariate	
	*p*	Relative risk (95% CI)	*p*	Relative risk (95% CI)
pT (pT2–4/pT1)	**0.039**	2.7 (0.99–7.6)	**0.025**	3.2 (1.1–9.5)
Lymph node metastasis (positive/negative)	0.25	1.7 (0.66–4.5)		
Histological grade (3/1 + 2)	0.26	1.7 (0.68–4.0)		
ER (negative/positive)	0.21	1.8 (0.73–4.4)		
PR (negative/positive)	**0.032**	2.9 (1.0–7.9)	**0.0051**	4.0 (1.4–12)
HER2 (negative/positive)	**0.042**	3.6 (0.84–16)	**0.013**	4.9 (1.1–22)
Ki67 LI (≥ 20%/< 20%)	0.051	2.5 (0.97–6.2)		
Tim1 (positive/negative)	**0.0025**	6.2 (1.4–27)	**0.024**	4.3 (0.97–20)

*Note:* *p* < 0.05 was considered significant and examined in multivariate analysis and described as bold.

We next examined the relationship between Tim1 immunoreactivity and clinical outcomes in ER‐positive breast cancer cases that had received endocrine therapy. Tim1 immunoreactivity was significantly associated with shorter disease‐free survival in these patients (Figure 3c, *p* = 0.0012), whereas no significant correlation was observed in other patients (Figure 3d, *p* = 0.089). As shown in Table 4, multivariate analysis demonstrated that only Tim1 immunoreactivity was an independent prognostic factor for disease‐free survival in ER‐positive patients who received endocrine therapy (*p* = 0.013).

**Table 4:** Univariate and multivariate analysis of disease‐free survival in 94 ER‐positive breast carcinoma patients who had received endocrine therapy.

	Univariate		Multivariate	
	*p*	Relative risk (95% CI)	*p*	Relative risk (95% CI)
pT (pT2–4/pT1)	**0.0071**	4.1 (1.5–12)	0.19	2.1 (0.68–6.5)
Lymph node metastasis (positive/negative)	0.051	2.8 (1.0–7.7)		
Histological grade (3/1 + 2)	**0.031**	3.7 (1.3–11)	0.58	0.67 (0.17–2.7)
PR (negative/positive)	**0.022**	3.6 (1.3–10)	0.15	2.4 (0.75–7.3)
HER2 (negative/positive)	0.87	0.88 (0.20–3.9)		
Ki67 LI (≥ 20%/< 20%)	**0.0065**	4.5 (1.6–12)	0.17	2.7 (0.69–10)
Tim1 (positive/negative)	**0.0007**	8.1 (1.8–36)	**0.013**	5.6 (1.2–27)

*Note:* *p* < 0.05 was considered significant and examined in multivariate analysis and described as bold.

## 4. Discussion

Here, we investigated the clinicopathological significance of Tim1 and its association with therapeutic resistance in human breast carcinoma tissues using immunohistochemistry. Tim1 immunoreactivity was predominantly detected in the cytoplasm and membrane of breast carcinoma cells, whereas it was negligible in normal breast epithelium, suggesting that Tim1 may play a pivotal role in breast cancer progression. In the present study, Tim1 immunoreactivity was significantly correlated with pT, lymph node metastasis, histological grade, and Ki‐67, a marker of cell proliferation. These findings indicate that Tim1 is highly expressed in breast cancers with an aggressive phenotype characterized by increased invasive and proliferative activity. Consistently, high Tim1 expression in gliomas has been reported to correlate with Karnofsky Performance Score and histological grade [17]. Tim1 has been shown, through in vitro studies, to promote cell proliferation, migration, and invasion in various types of cancer. For example, previous studies have demonstrated that Tim1 activates PI3K/AKT signaling in lung cancer, cervical cancer, and glioma cells [11, 13, 16], and MEK/ERK signaling in gastric cancer [10, 14], thereby promoting tumor malignancy. Activation of the PI3K/AKT and MEK/ERK pathways is closely associated with breast cancer progression through the regulation of various cellular processes, including the cell cycle, cell migration, and EMT. Furthermore, knockdown of Tim1 in glioma cells suppressed the expression of cytokines such as transforming growth factor‐*β*1 (TGF‐*β*1), interleukin (IL)‐6, IL‐4, and IL‐10, and activation of the PI3K/AKT pathway partially reversed these effects [13]. These cytokines, especially TGF‐*β* and IL‐10, are well known to induce both the malignant transformation of cancer cells and immunosuppressive effects in macrophages, thereby facilitating tumor progression. Taken together, Tim1 may not only directly enhance the proliferation and migration of breast cancer cells but also drive breast cancer progression by modulating both tumor cells and the tumor microenvironment through cytokine secretion, mediated via the PI3K/AKT and MEK/ERK signaling pathways. However, it should be noted that this study is limited by the lack of direct evidence regarding the biological function of Tim1 in breast cancer cells. Although immunohistochemical analysis of clinical specimens suggests that Tim1 expression may be associated with poor prognosis, and previous studies in other cancer types have indicated a role for Tim1 in tumor progression via the PI3K/AKT and MEK/ERK signaling pathways, these mechanisms have not been directly demonstrated in breast cancer. Therefore, further studies are warranted to elucidate the underlying molecular mechanisms using in vitro cell line models and in vivo mouse models.

On the other hand, this study identified a significant correlation between Tim1 expression and lymph node metastasis, whereas Diniz et al. reported no such association in breast cancer tissues based on immunohistochemical analysis [18]. However, the present study focused exclusively on invasive ductal carcinoma, whereas previous reports included other subtypes such as invasive lobular and papillary carcinoma, which may explain the discrepancy in results. Clarifying the clinical significance of Tim1 expression in a large cohort encompassing diverse breast cancer diagnoses will provide deeper insights into Tim1 function. Other possible reasons for the discrepancy between the results of the two studies include differences in the antibodies used. Furthermore, whereas Diniz et al. employed a tissue microarray approach, the present study evaluated whole tissue sections, enabling a more comprehensive evaluation. In addition, in the previously reported cohort, only 6.5% of cases were classified as histological Grade 1, whereas in our study, Grade 1 tumors accounted for 38.8% of cases, suggesting that Tim1 may also play an important role in low‐grade tumors.

Tim1 expression was significantly correlated with shorter disease‐free survival in breast cancer patients, consistent with findings in other malignancies such as lung cancer, gastric cancer, and glioma [10, 11, 17, 25], and was identified as an independent prognostic factor for disease‐free survival by multivariate analysis. Notably, in patients who had received chemotherapy, Tim1 was associated with an increased risk of recurrence and remained an independent prognostic factor for disease‐free survival. Chemoresistance in breast cancer remains a major challenge, and extensive research has been conducted to clarify its underlying mechanisms [26, 27]. However, these mechanisms, including their association with Tim1, have not yet been fully elucidated. The anthracycline epirubicin and the taxanes paclitaxel and docetaxel are commonly used chemotherapeutic agents for breast cancer, and activation of the PI3K/AKT pathway by various factors in breast cancer cells contributes to resistance to these drugs [28–30]. Previous studies have also suggested a possible association between Tim1 and induction of EMT in gastric cancer cells [14], which is widely recognized as a crucial step in the acquisition of chemoresistance in breast cancers [31, 32]. Furthermore, Tim1 expression was identified as an independent prognostic factor for disease‐free survival in ER‐positive patients who had received endocrine therapy. Ectodomain shedding of Tim1 has previously been reported to be associated with activation of the IL‐6/STAT3/HIF‐1*α* axis in ccRCC‐derived cell lines [33]. Overactive HIF‐1*α* may partially compensate for impaired ER*α* function, such as during endocrine therapy. Previous studies have demonstrated that certain genes, including KDM4B, STC2, and VEGFA, contain both HIF‐1*α* response elements and estrogen response elements, and that ER*α*‐positive breast cancer cells transduced with HIF‐1*α* become more resistant to tamoxifen and fulvestrant [34]. These findings suggest that HIF‐1*α* may drive the expression of these genes to counteract tamoxifen‐mediated inhibition of ER*α* signaling. Therefore, Tim1 may contribute to endocrine therapy resistance via upregulation of HIF‐1*α*. Recently, a human monoclonal IgG1 antibody against the extracellular domain of Tim1 was developed, and an antibody–drug conjugate (CDX‐014) was generated by conjugating it to the cytotoxin monomethyl auristatin E [35]. CDX‐014 showed in vitro cytostatic and cytotoxic activity against Tim1‐expressing cell lines, and in a first‐in‐human trial in patients with advanced refractory RCC, it demonstrated antitumor activity, including one prolonged partial response and a clinical benefit rate of 31% [36]. These findings suggest that Tim1‐targeted therapies may also hold potential for breast cancer treatment. On the other hand, the number of cases included in this study was imbalanced, with 94 ER‐positive breast cancer patients receiving endocrine therapy compared with only 22 patients in the remaining group. Therefore, because the potential influence of differences in sample size cannot be excluded, the relationship between Tim1 expression and resistance to endocrine therapy needs to be further evaluated in a larger cohort with more balanced treatment backgrounds.

On the other hand, analyses using public databases such as TCGA have demonstrated that high Tim1 expression in tumor tissues is associated with worse prognosis in stomach adenocarcinoma and lung adenocarcinoma, whereas it is linked to better prognosis in bladder urothelial carcinoma, renal cell carcinoma, head and neck squamous cell carcinoma, and skin cutaneous melanoma [12], with no significant correlation observed in breast cancer. Tim1 is expressed not only in cancer cells but also in immune cells, primarily T cells, so its effects on tumors may vary depending on the expressing cell type. In addition, Tim1 has been reported to regulate tight junctions in colorectal cancer cells, thereby reducing adhesion and invasive potential, and serving as a favorable prognostic factor associated with prolonged disease‐free survival [37]. Tim1 interacts with PS exposed on apoptotic cells and extracellular vesicles, as well as binding to Tim4. In vitro assays have demonstrated that Tim1 on colorectal cancer cells can be activated by recombinant Tim4, and that such activation significantly increases the frequency of apoptotic cells via upregulation of FasL expression [38]. However, the effects of Tim1 binding to its ligands in cancer cells, as well as the differences in function depending on the specific ligand, remain largely unknown. For example, cell–cell interactions mediated by extracellular vesicles are known to play an important role in the tumor microenvironment. However, it remains unclear what effect Tim1 has on cancer cells through its involvement in the uptake of extracellular vesicles. Tim1 may exert distinct functions in cancer cells depending on the surrounding ligand context, warranting further investigation.

In summary, we demonstrated by immunohistochemistry that Tim1 expression is significantly correlated with the risk of recurrence in breast cancer patients, including those who received adjuvant chemotherapy or endocrine therapy. These findings suggest that Tim1 may be an important therapeutic target for improving treatment in breast cancer patients and could serve as a strong prognostic factor associated with therapeutic resistance.

## Author Contributions

**Mio Yamaguchi-Tanaka:** conceptualization, formal analysis, funding acquisition, investigation, visualization, writing – original draft. **Kiyoshi Takagi:** conceptualization, data curation, project administration, supervision, writing – review & editing. **Ai Sato:** data curation, supervision. **Mai Sawafuji:** formal analysis, investigation, visualization. **Kanoko Nakamura:** investigation. **Minoru Miyashita:** resources. **Takashi Suzuki:** resources, supervision. **Yasuhiro Miki:** supervision.

## Funding

This study was supported by Japan Society for the Promotion of Science (10.13039/501100001691) (25K18736, 23K19493).

## Ethics Statement

All procedures performed in studies involving human participants were in accordance with the ethical standards of the institutional committee (Tohoku University Graduate School of Medicine) and with the 1964 Helsinki declaration and its later amendments or comparable ethical standards. Informed consent is not obtained because the design of the present study is a retrospective study.

## Conflicts of Interest

The authors declare no conflicts of interest.

## Data Availability

The data that support the findings of this study are available from the corresponding author upon reasonable request.
